# Retraction Note: Increased Gut Redox and Depletion of Anaerobic and Methanogenic Prokaryotes in Severe Acute Malnutrition

**DOI:** 10.1038/s41598-023-44597-3

**Published:** 2023-10-30

**Authors:** Matthieu Million, Maryam Tidjani Alou, Saber Khelaifia, Dipankar Bachar, Jean-Christophe Lagier, Niokhor Dione, Souleymane Brah, Perrine Hugon, Vincent Lombard, Fabrice Armougom, Julien Fromonot, Catherine Robert, Caroline Michelle, Aldiouma Diallo, Alexandre Fabre, Régis Guieu, Cheikh Sokhna, Bernard Henrissat, Philippe Parola, Didier Raoult

**Affiliations:** 1 Aix-Marseille Université, Unité de Recherche sur les Maladies Infectieuses et Tropicales Emergentes, UM63, CNRS 7278, IRD 198, INSERM 1095, Marseille, France; 2 Hôpital National de Niamey, Niamey, Niger; 3 Centre National de la Recherche Scientifique, UMR 7257, 13288 Marseille, France; 4 Aix-Marseille Université, Architecture et Fonctions des Macromolécules Biologiques, 163, avenue de Luminy, 13288 Marseille cedex 9, France; 5 Laboratoire de Biochimie, hôpital de la Timone, UMR MD2, IRBA, Marseille, France; 6 Institut de Recherche pour le Développement (IRD), URMITE, Dakar, Sénégal; 7 Service de Pédiatrie Multidisciplinaire, hôpital de la Timone Enfant, APHM, Marseille, France; 8 UMR_S 910, Aix-Marseille Université, Marseille, France; 9 Department of Biological Sciences, King Abdulaziz University, Jeddah, Saudi Arabia

Retraction of: *Scientific Reports* 10.1038/srep26051, published online 17 May 2016

Editors have retracted this Article.

After publication of this paper concerns about ethical oversight of this study were brought to the attention of the Editors. The Authors were not able to provide documentation of appropriate approval from an ethics committee in either Niger or Senegal, where the participants in this study were based.

Catherine Robert agrees with this retraction. Matthieu Million, Jean-Christophe Lagier, Souleymane Brah, Cheikh Sokhna, Philippe Parola, and Didier Raoult disagree with this retraction. Caroline Michelle and Bernard Henrissat did not explicitly state whether they agree with this retraction. Maryam Tidjani Alou, Saber Khelaifia, Dipankar Bachar, Niokhor Dione, Perrine Hugon, Vincent Lombard, Fabrice Armougom, Julien Fromonot, Alexandre Fabre, and Régis Guieu did not respond to correspondence from the Editors about this retraction. The Editors were not able to confirm current contact details for Aldiouma Diallo.

